# Hearing health care access for adult cochlear implant candidates and recipients: Travel time and socioeconomic status

**DOI:** 10.1002/lio2.1010

**Published:** 2023-01-17

**Authors:** Amanda G. Davis, Kelli L. Hicks, Margaret T. Dillon, Andrea B. Overton, Noelle Roth, Margaret E. Richter, Matthew M. Dedmon

**Affiliations:** ^1^ Division of Speech and Hearing Sciences, Department of Allied Health Sciences University of North Carolina at Chapel Hill Chapel Hill North Carolina USA; ^2^ Department of Otolaryngology/Head & Neck Surgery University of North Carolina at Chapel Hill Chapel Hill North Carolina USA; ^3^ Department of Audiology UNC Health Chapel Hill North Carolina USA

**Keywords:** cochlear implantation, demographics, disparities, geographic location

## Abstract

**Objectives:**

Access to cochlear implantation may be negatively influenced by extended travel time to a cochlear implant (CI) center or lower socioeconomic status (SES) for the individual. There is a critical need to understand the influence of these variables on patient appointment attendance for candidacy evaluations, and CI recipients' adherence to post‐activation follow‐up recommendations that support optimal outcomes.

**Methods:**

A retrospective chart review of adult patients referred to a CI center in North Carolina for initial cochlear implantation candidacy evaluation between April 2017 and July 2019 was conducted. Demographic and audiologic data were collected for each patient. Travel time was determined using geocoding. SES was proxied using ZCTA‐level Social Deprivation Index (SDI) information. Independent samples *t* tests compared variables between those who did and did not attend the candidacy evaluation. Pearson correlations assessed the association of these variables and the duration of time between initial CI activation and return for first follow‐up visit.

**Results:**

Three hundred and ninety patients met the inclusion criteria. There was a statistically significant difference between SDI of those who attended their candidacy evaluation versus those who did not. Age at referral or travel time did not show statistical significance between these two groups. There was no significant correlation with age at referral, travel time, or SDI with the duration of time (days) between initial activation and the 1‐month follow‐up.

**Conclusions:**

Our findings suggest that SES may influence a patient's ability to attend a cochlear implantation candidacy evaluation appointment and may further impact the decision to pursue cochlear implantation.

Level of evidence: 4 – Case Series.

## Graphical abstract

Differences in age, socioeconomic status (SES), and travel time to the clinic were compared for adults who were referred for cochlear implantation and either attended or did not attend the evaluation appointment and post‐activation appointments. There was a significant difference in SES between the groups, but not for age or travel time to the clinic. These findings elucidate areas of potential intervention for cochlear implant teams to expand access to cochlear implantation.

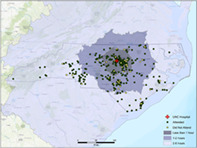

## 1 INTRODUCTION

Hearing loss is a growing public health concern, with an estimated one out of four people (23%) ages 12 years and older experiencing some degree of clinically significant hearing loss.^1^ Of those, an estimated 2.4 million individuals over 60 years of age could benefit from cochlear implant (CI) use^2^; however, less than 5% of adults who meet candidacy criteria have undergone cochlear implantation.^3^ Rates of CI utilization are likely impacted in part by inequitable access to hearing health care services, such as differences in travel time to a CI center or individual socioeconomic status (SES). There is a critical need to understand the potential influence of these variables on whether a patient referred for a cochlear implantation candidacy evaluation schedules and attends the appointment and whether a CI recipient adheres to the post‐activation follow‐up recommendations that support optimal outcomes with a CI.

The journey to cochlear implantation is not necessarily linear, and determination of candidacy for cochlear implantation requires a breadth of resources that are not limited solely to insurance coverage. The process requires several appointments with various medical providers to determine candidacy from both a medical and audiological perspective, surgical suitability, and other preoperative considerations.^4^ These appointments place high demand on patients' resources, including time, finances for travel and/or childcare, and the ability to take time (days or hours) off work. For example, Bush et al. investigated factors that deter a family from scheduling and attending a cochlear implantation evaluation for pediatric patients and found that increased distance from a clinic and rural classification were correlated with a delay in cochlear implantation candidacy determination and surgical follow‐up.^5^ Similarly, Hixon et al. reported delays in surgical intervention for adults living in rural communities, with increased drive times, decreased SES, and increased percentage of individuals with Medicaid insurance.^6^

Demands on resources persist into post‐operative care as well, with multiple recommended follow‐up intervals during the first year after implantation. At present, the recommended follow‐up intervals for adult CI recipients include initial activation of the device, then subsequent follow‐up visits at 1–2 weeks, 1 month, 3 months, 6 months, and 12 months post‐activation.^7^, ^8^ Delaying or not attending these follow‐up visits, particularly within the first three months post‐activation, may negatively influence speech recognition with the CI.^7^ The factors that can negatively impact an individual's timing of cochlear implantation evaluation could potentially pervade into their ability to adhere to the recommended follow‐up intervals, possibly inhibiting performance with the CI. To improve equity in access to cochlear implantation, there is a need to better understand the factors that may contribute to the ability to attend the candidacy evaluation and follow‐up appointments for adult CI candidates and recipients.

The present study examined the variables that may influence attendance to the cochlear implantation candidacy evaluation for referred patients and adherence to the recommended post‐activation visits. The reviewed variables included age at the time of referral, travel time to the CI center, and SES. The aim of the study was to elucidate potential barriers in access to cochlear implantation and post‐activation management to strategize efforts to improve access to hearing health care in our region. The findings presented here contribute to the limited body of research concerning disparities in access to cochlear implantation.

## 2 METHODS

A retrospective review was conducted to assess the patterns of attendance for referred patients to the cochlear implantation candidacy evaluation and adherence to recommended post‐activation visits as a function of age, travel time to the CI center, and SES. The procedures were approved by the study site's Institutional Review Board (IRB#: 09‐2328).

The query included data for adults referred to the CI center for their first cochlear implantation candidacy evaluation between April 2017 and July 2019. Patients in consideration for revision surgery or second‐side evaluation were excluded to control for additional factors that may influence attendance to the cochlear implantation evaluation appointment and adherence to the recommended post‐activation follow‐up. More recent data (i.e., after 2019) were excluded due to the confounding nature and implications of variables introduced by the COVID‐19 pandemic. The query was limited to data for patients residing in North Carolina for consistency of criteria used to determine county‐level socioeconomic and geographic data. The query included the following data: age at time of referral, insurance/payer type, zip code, county, and referral source (e.g., primary care physician, ENT physician, audiologist). For the patients who attended the cochlear implantation candidacy evaluation, the type of candidate was grouped by the audiologic findings, including conventional criteria (i.e., bilateral moderate‐to‐profound sensorineural hearing loss [SNHL]), expanded indications (i.e., unilateral moderate‐to‐profound SNHL or asymmetric SNHL), or not a candidate. If surgery was pursued, the duration of time between device activation and the initial post‐activation follow‐up visit was calculated. Patients who did not attend the initial evaluation were classified as “Did Not Attend” and were not grouped based on audiologic findings. Patients who met the cochlear implantation candidacy criteria and decided not to pursue cochlear implantation were classified as “Did Not Pursue” and by their respective audiologic candidate type.

### 2.1 Travel time

Travel time to the CI center was calculated using geocoding, which is the process of using text‐based descriptions of locations (e.g., city, zip code) to create geographic coordinates for mapping and analysis. Travel time was preferred over a determination of miles away to the CI center as it accounts for both location and the road network. Travel time was calculated in ArcGIS 10.8.1 software^9^ with the Closest Facility tool, using the StreetMap Premium North America HERE (2017, Release 3) road network data. Car‐based travel and optimal driving conditions (e.g., no traffic) were assumed. The Service Area tool (ArcGIS 10.8.1) was used to incorporate the areas that can be reached in a specified amount of time using a given road network, which accounts for the density of the input road network.

### 2.2 Socioeconomic status

SES was proxied using the Social Deprivation Index (SDI) as originally developed by Butler et al. and updated by the Robert Graham Center.^10^, ^11^ The SDI is a composite measure of the following variables for each aggregated Zip Code Tabulation Area (ZCTA): percentage of people living in poverty, single‐parent households, rented housing unit, or an overcrowded housing unit; percentage of people with less than 12 years of education; percentage of households without a car; and percentage non‐employed adults under 65 years of age.^11^ The SDI considers the intersectional nature of multiple variables that can contribute to an area's SES. The scale ranges from 1 to 100, with higher numbers indicating higher levels of deprivation, and thus a lower SES.

### 2.3 Data analysis

The first aim of this study was to assess the variables that may influence whether a referred patient schedules and subsequently attends a cochlear implantation candidacy evaluation. Independent samples *t* tests compared age at time of referral, travel time to the CI center, and SES (SDI) between those who did and did not schedule/attend the cochlear implantation candidacy evaluation, using the SPSS statistical software (version 27).

The second aim was to assess whether age, travel time, and SES via SDI influenced the timeline to return for the recommended post‐activation follow‐up visits. At the study site, the recommendation is to return one month after initial CI activation for assessment and mapping. Pearson correlations assessed the association of these variables and the duration of time between initial CI activation and when the patient returned for their first follow‐up visit.

## 3 RESULTS

Demographic information was available for 390 patients of the 394 total patients who met the criteria for inclusion. Table 1 lists the demographic information for the referred patients who did versus did not attend the cochlear implantation candidacy evaluation, including age, travel time, insurance type, SDI, candidacy determination, and decision regarding implantation.

**TABLE 1:** Demographic information for the patients who were referred for a cochlear implant candidacy evaluation and either attended or did not attend the appointment

	Attended (*n* = 349)	Did not attend (*n* = 41)
Mean age at time of referral (yrs)	63 (SD: 17)	60 (SD: 19)
Mean travel time (min)	82 (SD: 56)	87 (SD: 47)
Insurance/payer type (%)	Private insurance: 138 (39.5%)	Private insurance: 10 (24.3%)
	Medicare: 125 (35.8%)	Medicare: 19 (46.3%)
	Medicaid: 23 (6.5%)	Medicaid: 7 (17.0%)
	Private Medicare: 51 (14.6%)	Private Medicare: 5 (12.1%)
	Tricare: 7 (2.0%)	Tricare: 0 (0%)
	Other: 5 (1.4%)	Other: 0 (0%)
Mean SDI	49 (SD: 26)	59 (SD: 25)
Referral source	ENT: 224 (64.1%)	ENT: 29 (70.7%)
	Primary care: 34 (9.7%)	Primary care: 7 (17.0%)
	AuD: 56 (16.0%)	AuD: 3 (7.3%)
	Self: 28 (8.0%)	Self: 0
	No information available: 7 (2.0%)	No information available: 2 (4.8%)
Type of candidate	Conventional: 195 (55.8%)	Unknown
	Expanded indications: 79 (22.6%)	
	Non‐candidate: 75 (21.4%)	
Elected to not pursue cochlear implantation	Total: 111 (31.8%)	–
	*Conventional: 64* (*57.6%*)	
	*Expanded indications: 42* (*37.8%*)	
	*Evaluation discontinued: 5* (*4.5%*)	

Abbreviations: AuD, audiologist; ENT, Ear, Nose, & Throat physician; SD, standard deviation; SDI, Social Deprivation Index; yr, year.

### 3.1 Factors influencing cochlear implantation evaluation attendance

**FIGURE 1:**
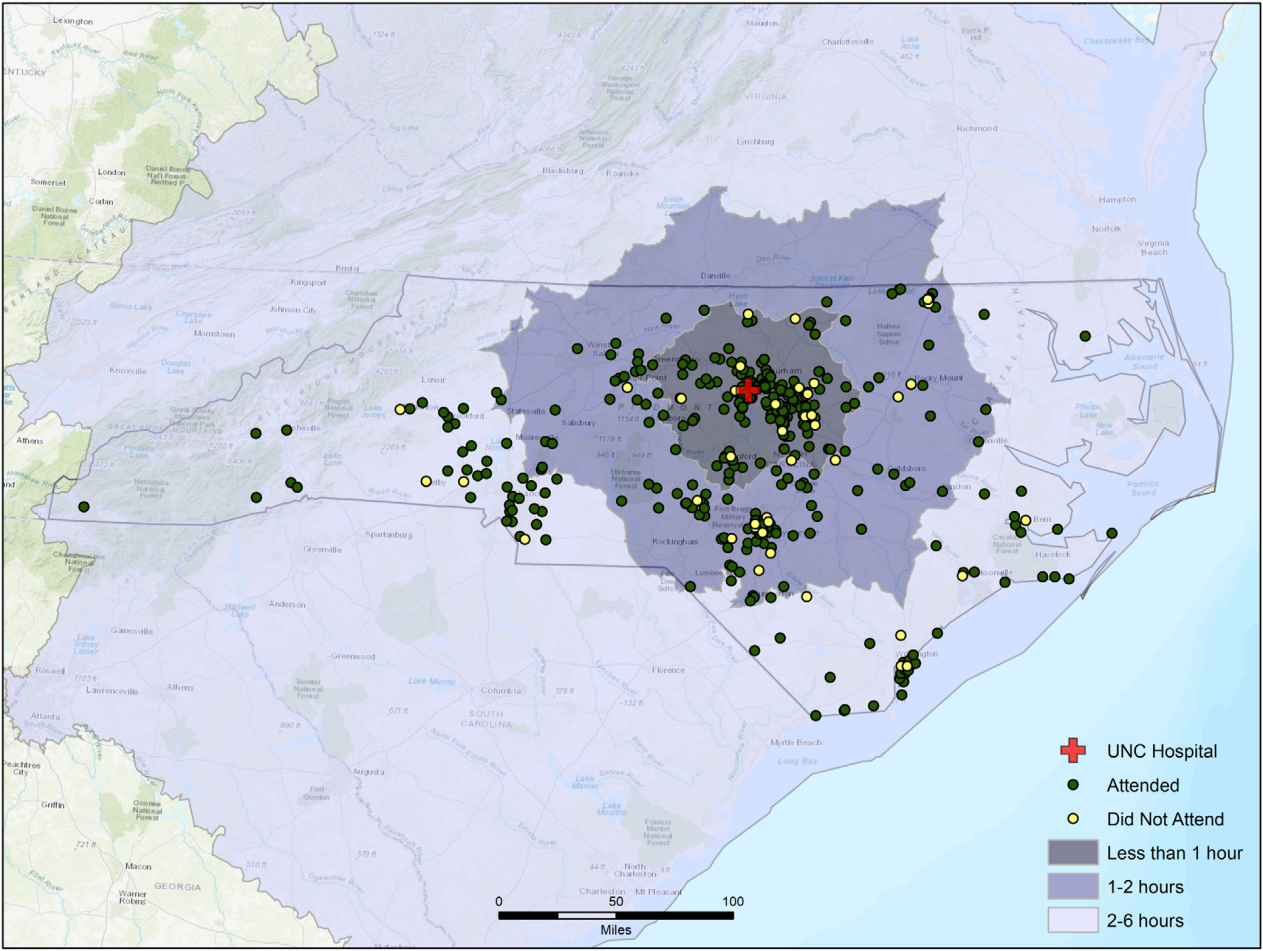
Map of the travel time to the cochlear implant center and whether the referred patient attended (green circles) versus did not attend (yellow circles) the cochlear implantation candidacy evaluation. Shading indicates the travel time, as defined in the legend.

Of the 390 patients, 41 (11%) did not schedule or attend the candidacy evaluation. For those who attended versus did not attend, the mean age at referral was 63 years (SD: 17 years) versus 60 years (SD: 19 years), travel time was 82 min (SD: 56 min) versus 87 min (SD: 47 min), and SDI 49 (SD: 26) versus 59 (SD: 25), respectively. Figure 1 illustrates the geospatial distribution and drive time of patients categorized by whether they attended or did not attend their cochlear implantation evaluation appointment. The shaded region represents the drive time with lighter shading indicating longer drive times. There was a significant difference between those who attended versus did not attend on SDI (*t*_(380)_ = −2.2, *p* = .031). The two groups, those who attended and versus those who did not attend, did not differ significantly on age at referral or travel time to the CI center (*p* ≥ .222).

### 3.2 Factors influencing the timeline for post‐activation follow‐up

Of the 349 who presented to the CI center for a candidacy evaluation, 155 underwent cochlear implantation during the reviewed period. Of those, the mean age at evaluation was 63 years (SD: 17 years), the mean travel time was 90 min (SD: 58 min), and the mean SDI was 49 (SD: 26). There was no significant correlation with the duration of time (days) between initial device activation and the 1‐month follow‐up for age at referral (*r* = −.14, *p* = .080), travel time to the CI center (*r* = −.01, *p* = .889), or SDI (*r* = .01, *p* = .87).

## 4 DISCUSSION

The decision to pursue cochlear implantation involves a multitude of factors and considerations on part of the patient. These considerations begin with the ability, both financially and geographically, to receive an evaluation at a CI center. The current study highlights the ways in which these factors contribute to patients' ability to attend their evaluation appointment and offers pause for commentary on behalf of those who did not. Given documented disparities in access to hearing health care across rural and urban settings,^6^ race,^12^, ^13^ and educational background,^14^ it is anticipated that SES and geographic location in relation to the CI center may also present barriers to timely hearing health care.

Such accessibility challenges permeate the hearing health care system from initial hearing evaluation,^15^, ^16^ to hearing aid access and utilization,^17^, ^18^ and into cochlear implantation.^6^, ^19^ Each of these steps acts as an essential component to obtaining a CI, and barriers along this pathway may confound and deter from patient success. The influence of geographic distance to a CI center or SES on whether a referred patient attends a cochlear implantation candidacy evaluation and whether CI recipients attend the recommended follow‐up visits is not well understood. The present study investigated the influence of age, travel time, and SES on whether a referred patient attended the cochlear implantation evaluation and on the timeline CI recipients returned for the first recommended post‐activation visit. For the cochlear implantation evaluation, there was a significant difference between the SDI for the group of patients who attended versus did not attend the evaluation appointment, but no significant differences between groups for age or travel time. For the first recommended follow‐up visit, there was no significant correlation for time to return for follow‐up and age, travel time, or SDI. These data suggest that SDI may create a barrier to accessing cochlear implantation evaluation appointments but may not significantly impact those who elect to pursue cochlear implantation.

The intersection of SES and geographic location is high in North Carolina. More rural counties are further from the CI center, and there is a higher likelihood of lower SES. This conundrum exacerbates strain on resources, as drive times are increased, yet access to financial resources for transportation are more limited. Documentation of reasoning for appointment non‐attendance was inconsistently found in provider reports, but transportation was frequently reported as a barrier to appointment attendance when documentation did occur. Financial barriers could include the ability to pay for transportation costs (e.g., gas, vehicle upkeep, ridesharing, etc.), ability to take more time off from work, and ability to pay for adequate childcare, if needed. Impact of these barriers resulting from lower socioeconomic status may still be felt regardless of proximity to the clinic.

**FIGURE 2:**
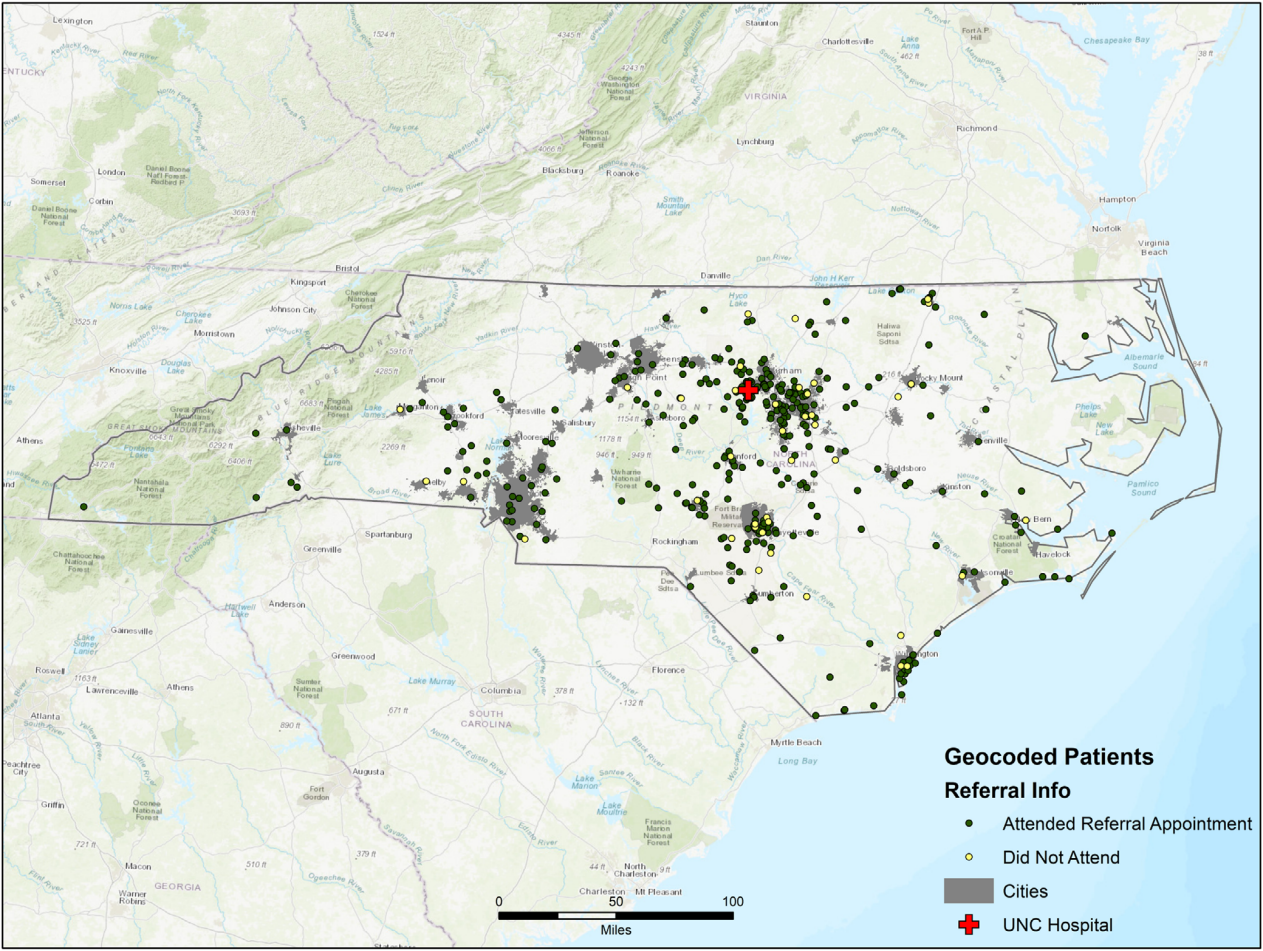
Geospatial distribution of patients referred for cochlear implantation evaluation with overlayed urban classification across North Carolina. Patients are labeled based on whether the referred patient attended (green circles) versus did not attend (yellow circles) the cochlear implantation candidacy evaluation. Shading indicates city classification—based on geographical area, population, and density—as defined in the legend.

The groups of patients who attended versus did not attend the cochlear implantation evaluation did not differ significantly on travel time. These results contradict those reported by Nassiri et al., who reported a significant travel burden in pursuing cochlear implantation for patients from rural settings that was associated with older age at implantation.^20^ This difference may be due to the distribution in urban versus rural areas in North Carolina. Figure 2 illustrates the distribution of patients who did or did not attend their referral appointment on a map, with the shading indicating an urban area. The Raleigh‐Durham area had the highest density of referrals, followed by Fayetteville, Charlotte, Wilmington, and Winston‐Salem, all urban areas. The Raleigh‐Durham area also had the highest density of patients who attended their referral appointment. This information is critical to note, as these areas are highly urbanized and are generally lower in SDI, indicating a generally higher economic status. The individuals driving from such urban areas, despite being further in distance, may not face the same barriers to care that would otherwise be exacerbated by increased geographic distance from the CI center in a more rural area. Additionally, patients coming from further distances may perceive higher stakes in the appointment, increasing their likelihood of attendance. Areas of future exploration will include a better understanding of the types of candidates referred by geographic location to assess how assurance of candidacy may influence appointment attendance.

We did not observe a significant relationship between the timeline for the first post‐activation follow‐up interval and the reviewed variables, which may have been due to biases within the sample. For example, the mean SDI of patients who returned for the follow‐up intervals was 49, which is the same as that of the patients who attended their evaluation appointment. Patients who have the ability to pursue cochlear implantation may have successfully circumvented barriers to receiving care, or may feel less of an impact of SDI. These factors may allow them to continue to receive CI care throughout the recommended follow‐up period. Additionally, this finding further suggests that SDI may play a role in a patient's decision to move forward with cochlear implantation.

While the present data are compelling as to the variables that may create barriers to accessing hearing health care, there are limitations worth consideration. One consideration of the present data is that the evaluative process for cochlear implantation typically begins with a referral from a medical provider. This assumes that local providers have the most recent information on candidacy criteria. Research suggests that patients who may benefit from cochlear implantation are under‐referred by primary care physicians and hearing health care professionals.^21^ It is unclear how this may bias the present data. The present data were also used by the clinical team for targeted outreach efforts to disseminate candidacy criteria information more effectively, potentially affecting patterns of referrals in future years. Further, the present sample uses county‐ and ZCTA‐level data that provides a broad glimpse into the macro‐level trends of patients who are referred for a cochlear implantation evaluation, but it may not provide a comprehensive representation of the challenges that individual patients may face. Also, while the present study had a large sample size, the retrospective nature of this study prohibited the exploration of individual considerations when electing to not pursue cochlear implantation. Future studies may benefit from a more qualitative study design focused on gaining this individualized perspective.

When considering trends in follow‐up after cochlear implantation, this study primarily focused on the first recommended post‐activation interval at the study site (i.e., 1 month). Attendance at later follow‐up intervals (e.g., >1 month) may also be influenced by socioeconomic and geographic variables. A patient's decision to pursue cochlear implantation may be influenced by the recommended follow‐up visits to the CI center within the first year, which also place strain on time and financial resources for the patient. A review by Ma et al. suggests that multiple follow‐up visits may not be necessary for every CI recipient considering when plateaus in speech recognition are observed.^22^ Additionally, some CI centers have investigated the use of remote programming,^23^ which could reduce the strain on the patient's time and financial resources. Future studies will aim to assess long‐term outcomes and patients' attitudes toward alternative service delivery models.

Future topics of interest will include a more robust look into the intersection of other factors that could be contributing to existing barriers to cochlear implantation, including insurance type, age, and access to transportation. Further endeavors will also aim to identify trends that may positively influence access to cochlear implantation candidacy, including updating referring providers about evolving candidacy criteria, streamlining the referral and evaluation process, implementing virtual components before the evaluation process to ease patient concerns about appointment attendance, and identifying regions for collaborative clinics to make follow‐up easier for patients in specific geographic regions. The identification of variables that clearly impede or enhance access to hearing health care will allow for a more targeted approach to ameliorating barriers to access to cochlear implantation from a systemic level.

## 5 CONCLUSIONS

Based on the data examined in this period, this project serves as an exploratory data analysis to guide future research on the topic of disparities in access to cochlear implantation candidacy evaluations. Our findings suggest that SES as proxied by SDI may influence a patient's ability to attend a cochlear implantation evaluation appointment at a major CI center and may further impact the decision‐making process in regard to pursuing a CI. Future research is necessary to understand the intersection of these variables, among others, on equitable access to timely CI intervention.

## CONFLICT OF INTEREST

MTD and MER are supported by a research grant provided to their university by MED‐EL Corporation. ABO serves on the Audiology Advisory Boards for Advanced Bionics and MED‐EL Corporation and is a consultant for Cochlear Corporation.
